# Spare the rod, spoil the child: measurement and learning from an intervention to shift corporal punishment attitudes and behaviors in Grenada, West Indies

**DOI:** 10.3389/fpubh.2023.1127687

**Published:** 2023-08-29

**Authors:** Barbara Landon, Elizabeth D. Thomas, Lauren Orlando, Roberta Evans, Toni Murray, Lauren Mohammed, Jesma Noel, Rashida Isaac, Randall Waechter

**Affiliations:** ^1^Windward Islands Research and Education Foundation, St. George’s, Grenada; ^2^Psychological Services Center, St. George's University, St. George’s, Grenada; ^3^Department of Public Health, St. George's University, St. George’s, Grenada; ^4^Department of Neuroscience, School of Medicine, St. George's University, St. George’s, Grenada

**Keywords:** corporal punishment, Caribbean, ECD scaling, parent attitudes, Measuring for Change, low- and middle-income countries

## Abstract

Childrearing practices in the Caribbean and other postcolonial states have long been associated with corporal punishment and are influenced by expectations of children for respectfulness and obedience. Evidence across settings shows that physical punishment of young children is both ineffective and detrimental. Saving Brains Grenada (SBG) implemented a pilot study of an intervention based on the Conscious Discipline curriculum that aimed to build adult caregivers’ skills around non-violent child discipline. We hypothesized that attitudes towards corporal punishment would shift to be negative as adults learned more positive discipline methods, and that child neurodevelopment would correspondingly improve. This report reviews the impact of monitoring and evaluation on the design and implementation of the intervention. Study 1 presents findings from the pilot study. Despite positive gains in neurodevelopmental outcomes among children in the intervention compared to controls, attitudes towards corporal punishment and reported use of it did not change. Additionally, several internal conflicts in the measures used to assess corporal punishment behaviors and attitudes were identified. Study 2 is a response to learning from Study 1 and highlights the importance for monitoring and evaluation to be data-informed, adaptive, and culturally appropriate. In Study 2, the SBG research team conducted cognitive interviews and group discussions with stakeholders to assess the content and comprehensibility of the Attitudes Towards Corporal Punishment Scale (ACP). This yielded insights into the measurement of attitudes towards corporal punishment and related parenting behavior, and prompted several revisions to the ACP. To accurately evaluate the intervention’s theory of change and its goal to reduce violence against children, reliable and appropriate measures of attitudes towards corporal punishment and punishment behaviors are needed. Together, these two studies emphasize the value of continuous monitoring, evaluation, and learning in the implementation, adaptation, evaluation, and scaling of SBG and similar early childhood development interventions.

## Introduction

Corporal punishment–physical force used and intended to cause pain or discomfort, however light (1)–is common worldwide (2). Despite its prevalence, and irrespective of cultural normativeness (3) or parental warmth during non-disciplinary interactions (4, 5), abundant evidence shows that corporal punishment,^1^ including spanking, is detrimental to young children’s development (4, 6–9) and ineffective at maintaining discipline (8–10). Children who are physically punished have fewer social skills, reduced empathy, more aggressive behaviors, lower achievement, higher rates of learning difficulties and physical and mental illness in both childhood and adulthood, and they are more likely to perpetuate violent behaviors as adults (11–14).

In the Caribbean, corporal punishment is considered a necessary part of childrearing (15–21). Due to a history of violence, danger or perceived danger, punitive attitudes and expectations for respectfulness and obedience, physical punishment has been passed from generation to generation as the most widely used disciplinary method (16–18, 21). Even recent studies in the region continue to document wide-scale prevalence of physical punishment: 88 percent of young adults in a Bahamian study reported having been hit or beaten (22), and 86 percent of adolescents in Suriname reported recurring harsh punishment (23). A review of UNICEF Multi Indicator Cluster Survey data of 5,339 mothers with children under age 5 across five Caribbean countries found that 57 percent used physical punishment (spanking) or harsh physical punishment (using an object, shaking, hitting on the face or head, beating) and 55% reported psychological aggression (screaming, yelling, calling the child names) (24).

Despite all Caribbean states having signed the UN Convention on the Rights of the Child (25), which commits them to protecting children from violence, including corporal punishment, only one nation, Cuba (26), has banned corporal punishment in all settings. Understanding why a known risk factor for child development persists is key to early childhood interventions (27). However, assessing beliefs and practices around corporal punishment is fraught with challenges. Measures generally rely on retrospective self-reports. Most researchers have used self-generated questionnaires or items from other measures (28), and most instruments, such as the Attitudes Towards Spanking Scale (29), the Parent–Child Conflict Tactics Scale (30), and the Punitive Discipline Scale (31), are U.S.-based and have not been validated in postcolonial societies or low- and middle-income countries (LMICs).

Attitudes towards corporal punishment are challenging to assess (27). Straus (9) and Kish and Newcombe (10) suggest that beliefs around corporal punishment are often based in selective inattention and an inability to recognize the potential harm. Lack of knowledge or confidence in alternative discipline strategies may also contribute to the difficulty of assessing attitudes (27, 32). For instance, most Caribbean parents believe that physical punishment is necessary and have traditionally used discipline to show disapproval of undesired behavior, rather than positive discipline to encourage desired behavior (24, 33).

Interventions aimed at reducing corporal punishment range from targeted programs for at-risk families to universal education programs to strengthen parenting skills and/or provide education on the harmful effects of punitive parenting (34). The World Health Organization (WHO) recommends evidence-based practices for reducing violence against children, including implementation of laws, addressing social norms and values, ensuring safe environments, providing caregiver support, and providing education and life skills (35). A recent analysis of interventions to reduce violence against children in LMICs concludes that most effective interventions have focused on education and life skills and addressing norms and values, although study confidence is medium to low, and geographic distribution of the research is uneven–no Caribbean studies are included (36).

Effective early childhood interventions are needed, including those that ensure responsive physical and social–emotional care as a foundation for neurodevelopment (37). Nonetheless, challenges in improving access to early childhood development (ECD) interventions across populations persist (38–40). Efforts to scale interventions need accurate, well-integrated monitoring, evaluation, and learning (MEL) systems (41, 42) that assess effectiveness within an intervention’s particular context.

The Saving Brains Grenada (SBG) initiative was launched in 2014 in response to concerns about violence against women and children and calls for interventions that address ECD in LMICs (15, 43, 44). SBG is a physical punishment prevention program that promotes neurodevelopment by fostering social–emotional connections between caregiver and child (45, 46). The intervention focuses on teaching and modeling responsive caregiving behaviors under which young children thrive: physical and psychological safety and secure attachment. As children experience safety, adult composure, and consistent social–emotional connections, they are more cooperative and self-regulated, requiring fewer and less punitive disciplinary events (47). They are also more willing to explore their environments, which promotes neuronal growth and cognitive and social–emotional development (47, 48).

Consistent with WHO’s recommendations for addressing norms and values, ensuring safety, supporting caregivers, and providing education and life skills, we hypothesized that when adults acquire knowledge and skills about positive responsive care, create safe, predictable environments, and manage conflict and misbehavior effectively, their use of physical punishment will decline, and attitudes towards corporal punishment will become disapproving.

From the outset, SBG’s intention has been to learn from doing by piloting the initiative’s intervention program and its assessment tools for scale-up in the Caribbean and similar developing regions. This report presents two MEL studies from the SBG program. Study 1 presents findings from the 2014–2016 SBG pilot and highlights the importance for MEL to be data-informed and for interventions to be adaptive. Study 2 is a response to learning from Study 1, and describes findings and measurement revision to improve both assessment and intervention.

## Study 1: Pilot intervention

### Methods

The SBG pilot study was a parallel single-blind, waitlist-controlled, post-only design, enrolling children and their parents who were either participating or wait-listed in a community-based intervention (St. George’s University IRB #14099; https://clinicaltrials.gov/ct2/show/NCT04697134). The intervention was based on the Conscious Discipline curriculum (46, 49), which aims to build adult caregivers’ skills in non-violent child discipline. For implementation, SBG partnered with the Roving Caregiver Program, a home visiting service providing infant stimulation to at-risk Grenadian children ages 0–3 (50). Roving Caregivers underwent intensive training in Conscious Discipline. The aim of the intervention was to promote social–emotional connection between Roving Caregivers and parents, and between parents and their children. We hypothesized that attitudes towards corporal punishment, and use of physical punishment, would shift as parents learned positive discipline methods and their impact on a developing brain (51–53). A description of study methods and results is provided by Waechter and colleagues (45); a manual is available from the corresponding author. This case study specifically reports on methods and results of assessing attitudes and behaviors towards corporal punishment and parent–child interactions, rather than the effectiveness of the intervention, which is reported elsewhere (45).

As part of the SBG pilot, caregivers in intervention and control groups were administered questionnaires including the Home Observation for Measurement of the Environment (HOME, Part A) (54) Acceptance subscale and the Attitudes Towards Corporal Punishment (ACP) Scale (ACP; Appendix A). The HOME-A, a widely-used tool among ECD researchers, examines caregiver response to child behavior *via* parent report (reports no more than one instance of physical punishment in the past week) and assessor’s observation (caregiver not shouting at child, expressing overt annoyance with or hostility to child, hitting, scolding, or restricting child during home observation; Appendix B). The ACP was developed in-house to assess pre-post attitudes and behaviors related to corporal punishment after a literature search did not yield a suitable existing measure for the study setting. It adapted several items from the US-based Attitudes Towards Spanking Scale (29). The ACP includes items on physical punishment use and recency of use, as well as items assessed on a 5-point Likert-type scale of agreement.

Seven hundred fifty-two (752) families were enrolled in the SBG pilot, and were randomly selected for assessment. Three hundred forty-eight (348) participants were administered the HOME-A and ACP at baseline, with 379 administered post-intervention. Descriptives and chi-square analyses were used to compare groups on the ACP measure, and an independent samples t-test was used to compare groups on the HOME-A.

### Results

At both baseline and endline, in both arms, the vast majority of primary caregivers of 0–24 month-old children used physical punishment, and had done so in the past week (Table 1).

**Table 1:** Percentages of participants pre- and post-intervention who utilize corporal punishment.

	Pre-intervention (*n* = 348)		Post-intervention (*n* = 379)	
	Control	Intervention	Control	Intervention
Caregivers smack/beat their child	81%	84%	91%	96%
Caregivers smacked/beat their child within the last week	76%	81%	72%	73%

Significant differences between post-intervention and control groups were detected on responses to the HOME-A Acceptance subscale, t(583) = −2.09, *p* = 0.037. Intervention group caregivers demonstrated more acceptance of their child’s behavior (M = 3.52, SD = 3.42) compared to caregivers in the control group (M = 2.96, SD = 3.12). (Higher mean = greater acceptance of child behavior.) However, there were no significant differences between post-intervention and control groups on responses to the ACP. In both groups, most participants believed that corporal punishment helps build respect for authority figures (72–75 percent), helps children become successful adults (71 percent), and should be used as a disciplinary method in schools (59–67 percent). When considering differences detected between post-intervention and control groups on responses to the HOME-A Acceptance subscale, this lack of difference in the ACP suggested that the ACP may not have been effective in capturing differences between the groups, thereby raising questions about its validity and/or sensitivity.

**Figure 1:**
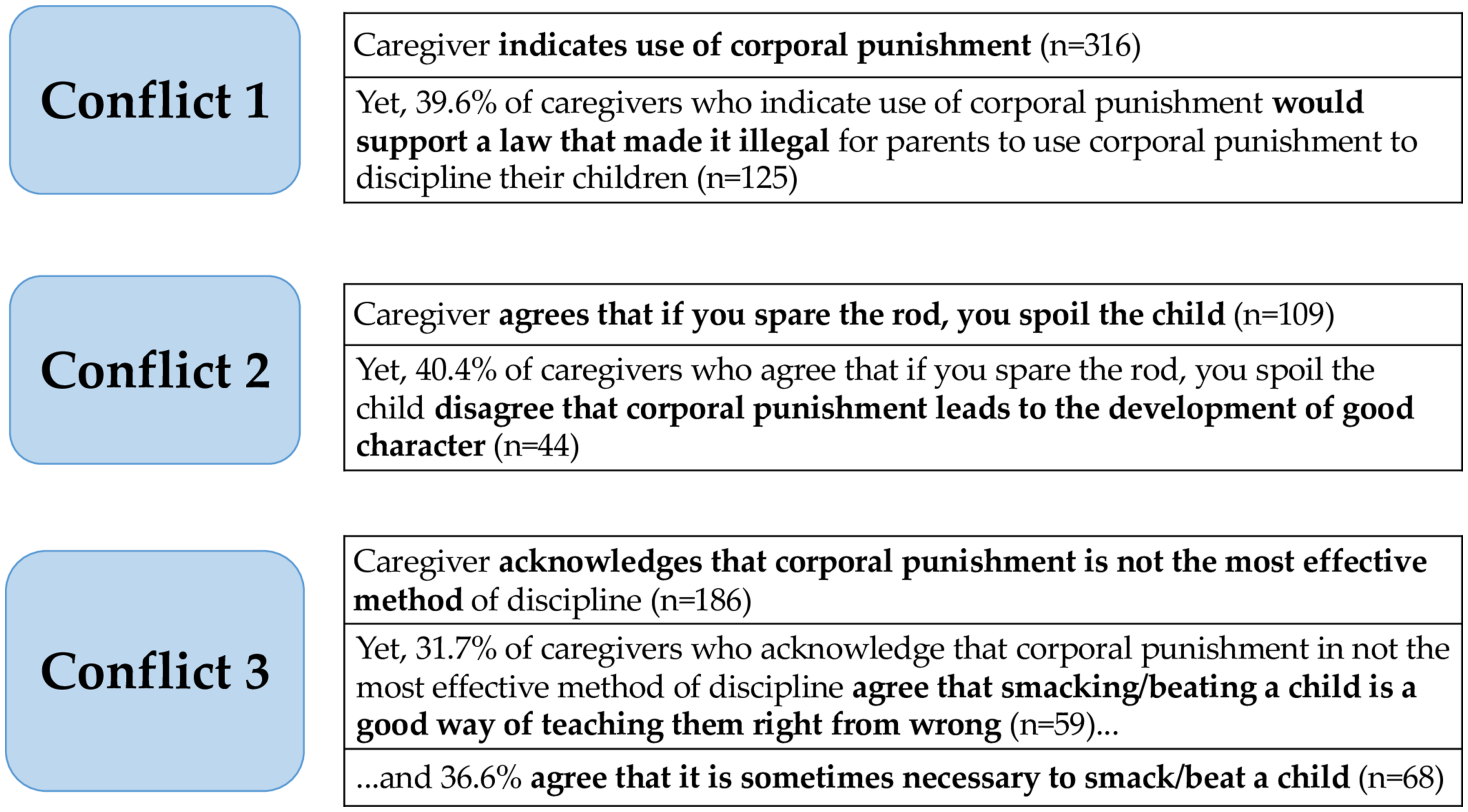
Conflicting responses found in the ACP results during the pilot study.

Further analysis of baseline ACP data yielded conflicts within participants’ responses. For example, of 316 participants who reported using corporal punishment, 125 (39.6 percent) also agreed with the statement, “I would support a law that made it illegal for parents to use corporal punishment.” Of the 109 participants who chose the statement, “I believe that if you spare the rod, you spoil the child” as most aligned with their views, 44 of those same participants (40.4 percent) disagreed with the statement “Corporal punishment leads to the development of good character.” Finally, of the 186 participants who reported that corporal punishment was not their most effective form of discipline, 31.7 percent (n = 59) nonetheless agreed with the item, “Smacking/beating children is a good way to teach them right from wrong,” or the item, “It is sometimes necessary to beat a naughty child” (36.6 percent, n = 68) (See Figure 1).

### Study 1 Discussion

We expected to see attitude changes towards corporal punishment among intervention participants, as measured by the ACP, and were surprised to find none. We were intrigued to find internal conflicts in participants’ responses on the ACP and incongruent findings between the ACP and HOME-A. One explanation for these discrepancies is that study participants misunderstood items or response options on the ACP. Another is that caregivers feel ambivalent about corporal punishment, resulting in conflicting responses. Payne (18) described a similar “interesting ambiguity” among survey respondents regarding use of corporal punishment in Barbados. Similar discrepancies between attitudes and behaviors exist elsewhere: mothers in 34 LMICs acknowledged using physical punishment, even though most said they believed physical punishment was unnecessary (27). Attitudes and practices around corporal punishment may also take time to shift–the study may have been of insufficient duration to demonstrate change.

Given the high number of participants endorsing and defending corporal punishment, SBG used ACP data from the pilot study to shift emphasis away from physical punishment. Rather, we drew from principles of Conscious Discipline (46) to focus on what was wanted—responsive care—rather than what was not wanted—physical punishment. Interventionists encouraged parents to add new, positive behaviors without challenging existing beliefs or practices. This change in intervention approach was a direct result of the SBG pilot study measurement system.

## Study 2: Quality assurance of the Attitudes Towards Corporal Punishment Scale

To evaluate SBG’s theory of change and its objective of reducing violence against children, reliable measurement of corporal punishment attitudes and behaviors is needed. In response to findings from Study 1, the SBG research team conducted a quality-assurance study of the ACP (Study 2) to explore whether the scale fulfills its intended purpose.

### Methods

From February to August 2022, the SBG team conducted group discussions and cognitive interviews to capture feedback on ACP items’ wording, content, and comprehensibility. Cognitive interviewing is a qualitative method that explores how people process and respond to questionnaires, with the goal of developing instruments that produce high quality data (55). Cognitive interviewing is a valuable method for developing and improving quantitative surveys, and adapting measures developed in one context and utilized in another–it can help identify and resolve issues with survey questions, including word choice and alignment with local views (56).

**Figure 2:**
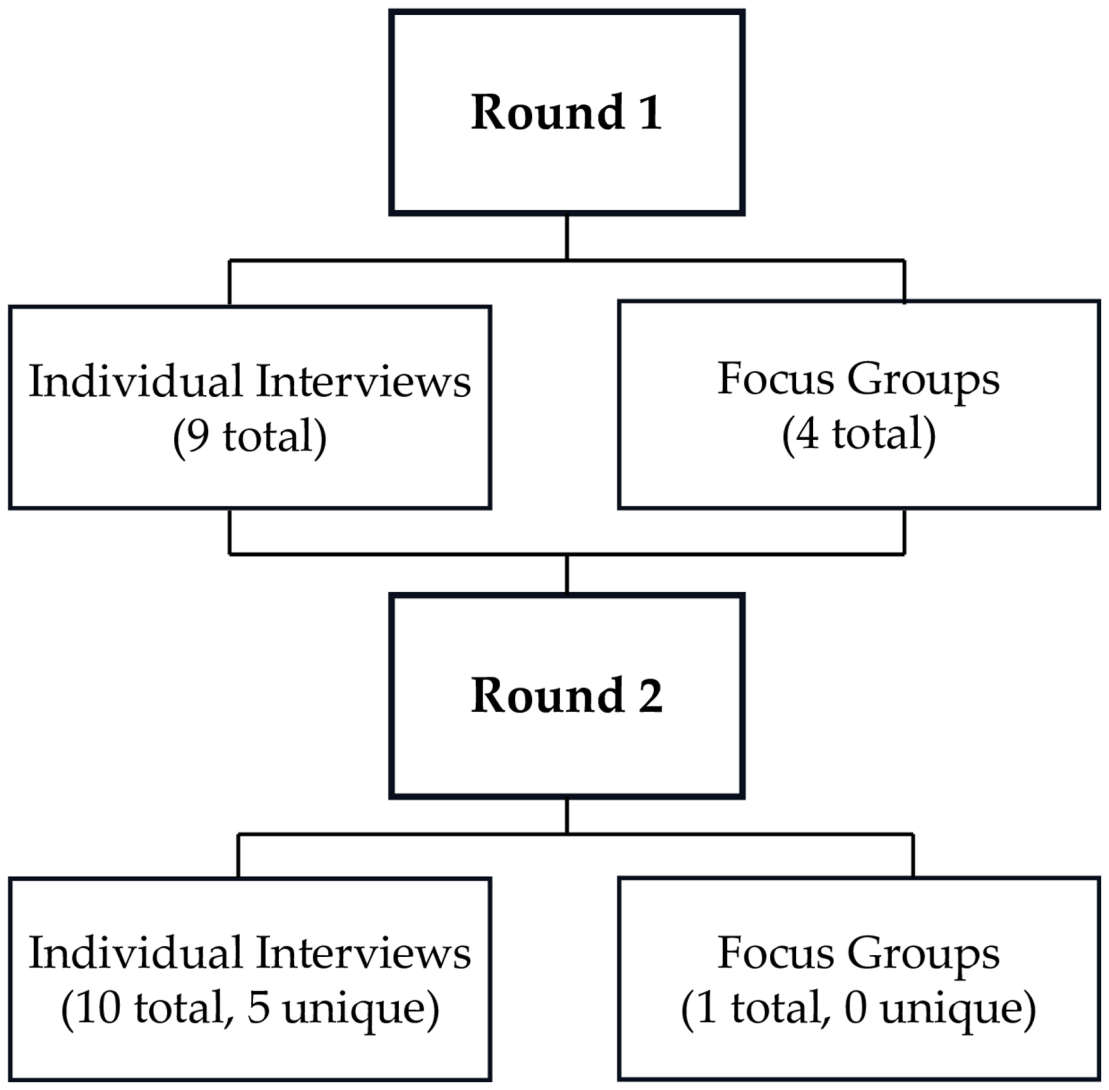
Cognitive interviewing process for ACP quality assurance study.

First, a group discussion was conducted with Roving Caregiver Program supervisors who oversaw implementation of the pilot. The SBG team proceeded with two rounds of cognitive interviewing and group discussions, using concurrent probing techniques to review each item and explore item clarity, wording, and response options. The team followed general guidance for conducting cognitive interviews to improve questionnaire design (57–59) and conducted two rounds of 9-10 interviews each (Figure 2). After the first round, team members reviewed all responses and wrote comments on each item. In the first round, the team recruited a convenience sample of parents from within the project’s network. In the second round, the team interviewed a subset of participants from the first round and also recruited new individuals from within the project network. In the second round, a revised ACP was used for all interviews (Appendix C). Interviews were conducted in-person or over video-conferencing (Zoom). All interviews were recorded, and detailed written notes taken. Responses were combined in a matrix for review.

Study 2 was exempt from IRB review given its focus on measure-testing quality assurance.

### Results

Four categories of issues with the ACP emerged: (1) definitions and measurement of corporal punishment; (2) restrictive items or response options; (3) unclear relationship between item and construct; and (4) minor issues with terminology and/or comprehension. For each theme, examples of the original item, findings from both rounds of cognitive interviews and group discussions, and modifications to items are outlined in Table 2; a table outlining findings for all items is available in Appendix D.

**Table 2:** Learning from the quality assurance of the ACP—examples of the original item, findings, and modifications.

Original item (Response option)	Findings from Round 1	Revised item (Response option)	Findings from Round 2
**Definitions and measurement of corporal punishment (behavior)**			
Have you ever smacked/beaten your child? (Yes/No) If yes, how recently? (Last week/Last month/Last 6 months/Last year) Please indicate in which circumstances, if any, you have smacked/beaten your child(ren) in the past year. (Open-ended)	- “Smack” and “beat” are different - Unclear how to answer if smacked but never beaten - This does not capture other forms of punishment - There is a difference between recency, frequency, and severity of punishment	Have you ever used any form of physical punishment with your child? (Yes/No) If yes, please describe. With hand? With object(s)? (Open-ended) When was the last time you used physical punishment? (Open-ended) How often do you use physical punishment? (Open-ended)	- Revision seems clear, but participants do not always clearly answer each part of the question
**Restrictive question/item or response options**			
Is corporal punishment an effective method of disciplining a child? (Yes/No)	- Participants are not always able to answer yes or no, several answer with “it depends” or “sometimes”	Physical punishment is an effective method of disciplining a child. (Agree/Disagree)*	- Revision seems clear, though item may not fully capture situation-dependent use of corporal punishment
**Minor issues with terminology**			
Smacking/beating a child is as unacceptable as hitting an adult. (Agree/Disagree)*	- “Just as bad” used instead of “unacceptable” in practice - Smacking, beating, and hitting all different terms	Using physical force on a child is the same as using physical force on an adult. (Agree/Disagree)*	- “Physical force” is too strong a term - General sense/purpose of the item seems clear

*Response options were a 5-point Likert-type response of Strongly Disagree, Disagree, Neutral, Agree, or Strongly Agree.

An additional topic that aligned with the study’s objective also came through in the data: conflicts between attitudes or beliefs and corporal punishment behavior. An overview of findings is presented here.

*(1) Definitions and measurement of corporal punishment.*

An early observation from participants was that the two descriptive terms used in the ACP to represent corporal punishment (“smack/beat”) were not considered equal forms of punishment.

*“It sounds like two different questions.”* -Roving Caregivers Supervisors Discussion

*“Lumping ‘smacked’ and ‘beat’ makes it difficult [to answer the question]*.” -Round 1, Group Discussion 3.

*“A tap/smack is using a hand or ruler across a leg or on the bottom…Beat is using force all over the body and it can cause bruising.”* -Round 1, Cognitive Interview 1.

In Round 1, participants were asked to provide their own definition of corporal punishment. Participants’ responses varied, with several mentioning physical punishment or naming a form of physical punishment (e.g., lashing); others described a broader definition, including other forms of punishment, such as “removing privileges” or “causing physical and emotional distress to a child/individual.”

The suggestion from participants to resolve this issue was to use a separate term, like “hit,” or to say “physical punishment” instead of naming a specific action. As a result, in Round 2, items previously asking about smacking/beating were revised to ask about physical punishment, and a definition of physical punishment was provided (see Appendix C).

The original version of the ACP included four items to record parents’ disciplinary actions (Appendix A, Items 1–4), including items asking if the participant had ever smacked/beaten their child(ren), and recency and circumstances of the event(s).

*“…You have to ask how often! ‘I beat them every day but not this morning.’”* -Round 1, Group Discussion 1.

In Round 1, participants indicated that this set of questions did not fully capture parents’ disciplinary actions, and the items were revised for Round 2 (Table 2).

*(2) Restrictive items or response options.*

In Round 1, several items were considered “too general,” and therefore difficult to answer. In addition, many of the items with yes/no response options in Round 1 were answered with “maybe” or “it depends.” Most participants felt that a dichotomous response option was too extreme or not sensitive enough. For example, the original ACP asked, “Does corporal punishment lead to the development of good character?” While some participants were able to respond “yes” or “no” with justification for their answers, others struggled:

*“I’m not sure. [It could go] both ways.”* -Round 1, Cognitive Interview 4.

For Round 2, these items (Appendix C, Items 11–15) were changed from questions with a dichotomous yes/no response option to statements with a 5-point agree/disagree Likert-type response. This change was well-received by those who participated in both rounds.

Two other items and their response options were considered restrictive. In one item, parents were asked to indicate the disciplinary method previously mentioned that had the most effect on a child’s behavior (Appendices A, C, Item 2). Participants, however, said that it depended on the child, the child’s age, the behavior that required punishment, and the parent themselves. At the same time, the SBG team wanted to better capture the potential conflict between parents’ disciplinary actions and what they perceive to work best; in Round 2, additional questions were added to explore this (Table 2; Appendix C).

The other item considered restrictive asked parents to identify, from among a list of statements on corporal punishment, the one that aligned best with their beliefs (Appendix A, Item 5; Appendix C, Item 4). Some participants felt that the statements were not mutually exclusive, or that multiple statements aligned with their beliefs.

*“They all apply to me. Growing up, my grandmother beat my older cousins, me too, we came up ok. For Option C (I'm comfortable with the idea of smacking/beating a child and will do it when I think it's necessary), right now technology has created a great wall. My grandma will watch you up and down. For me, children now lack discipline. I’m not against corporal punishment.”* -Round 1, Cognitive Interview 2.

Participants had suggestions for how to improve the content of each statement in the question, and each was revised ahead of Round 2. However, a few participants in Round 2 still felt the statements were not mutually exclusive, or representative of their beliefs.

*“I do not find any that represent me. (Interviewer then clarified to choose which comes closest to personal opinion). I don't like the idea of using physical punishment but I will sometimes use it; it’s something I don’t like to do but it does happen.”* -Round 2, Cognitive Interview 5.

A note for the participant above: she selected Option A, “I think it is always wrong to use physical punishment on a child,” as the one that best represents her, although Option B, “I do not like the idea of using physical punishment, but I will do it if nothing else works” fit more closely with her interview response.

For this item, participants also noted that the phrase “Spare the rod, spoil the child” (Option D) could be understood differently by different people, and could be a held belief alongside other statements in the item.

*“Option D, ‘“Spare the rod and spoil the child,’” is not the most intense…it could be both Options B and D.”* -Round 1, Group Discussion 1.

Responses to this item in Round 2 indicated that additional item generation and evaluation are needed.

*(3) Unclear relationship between item and construct.*

One item asked participants to agree or disagree with the statement, “Only bad parents smack/beat their children.” In Round 2, the statement was modified to change “smack/beat” to “physical punishment.” In both rounds, the statement was clear and participants were able to provide a response and a justification that aligned with their close-ended response.

*“Strongly disagree. It doesn't mean you're bad, you're making sure the child is on the right path. All parents mean well but circumstances and experience prevent them from being good. Willingness and support is needed.”* -Round 1, Cognitive Interview 6.

*“Agree, but parents might not be educated enough; they aren’t necessarily bad parents. They don’t mean to harm the child. Beating a child is just short term [effective]. Two or three days later, the child will do the same thing.”* -Round 2, Cognitive Interview 1.

In review of participants’ explanations, it was evident that responses were not reflective of an attitude towards corporal punishment, but rather an attitude towards parenting. It was also unclear how this item should be scored in a composite measure. As a result, the item was dropped from the scale.

*(4) Minor issues with terminology and/or comprehension.*

Cognitive interviewing and group discussions uncovered minor issues with terminology and/or comprehension (Table 2). For example, for the item, “Smacking/beating a child is as unacceptable as hitting an adult,” SBG team members who had administered the questionnaire noted that in practice they were saying “just as bad” instead of “as unacceptable,” so this change was made to the item. In Round 2, “smacking/beating” was replaced by “physical force” for this item, though participants felt the term was too strong and did not meet the objective of the item. Different terms and iterations of the item are being tested.

*(5) Conflicts between attitudes or beliefs and behavior.*

The SBG team identified several instances of conflict between attitudes or beliefs and participants’ actions (e.g., corporal punishment behavior), similar to those identified in Study 1.

For example, in response to the question about whether they had ever smacked/beaten a child, two participants said yes, but also mentioned negative outcome expectations. One participant, a grandmother, said she “threatens punishment to grandchildren, but it ruins the relationship.” Another participant said she smacked/beat her children, but “[did not] want [her] children to expect licks like [she herself had experienced], as it could “create a greater monster.”

As another example, in response to the item, “It is sometimes necessary to smack/beat a child,” agreement was not necessarily reflective of an endorsement of corporal punishment.

Interview notes: First answer given was “tend to agree” as they are “influenced by culture.” The second answer given was “strongly disagree” as it is “not necessary, you could use other methods.” -Round 1, Cognitive Interview 5.

*(6) Other findings.*

In a few cognitive interviews and group discussions, participants noted that the survey questions had been thought-provoking, suggesting that the process of responding to questions encouraged reflection of attitudes and beliefs around the practice:

*“It makes me think a lot more of other measures to try to correct behavior.”* -Round 1, Cognitive Interview 1.

*“Interesting. I never really thought about it so much before.”* -Round 2, Cognitive Interview 10.

## Discussion

These two studies share a common objective: improving and leveraging measurement and evaluation in order to strengthen an intervention. In Study 1, ACP results prompted a shift in focus to better align with local context. Moreover, conflicting results suggested a problem with measurement, the theory of change, or both. In Study 2, data from the ACP prompted critical revisions for future applications–results from cognitive interviews and group discussions yielded insights into the content and measurement of attitudes and behaviors. Together, the studies provide important learning for implementation, adaptation, evaluation, and scaling efforts to address physical punishment against young children.

### Key monitoring and evaluation learning

Findings from both studies suggest that, in the short-term, despite increased acceptance and tolerance for a young child’s behavior, attitudes towards physical punishment did not change as anticipated. Even as they learned and implemented methods for relationship-based positive discipline that supports neurodevelopment, parents continued to endorse cultural norms around corporal punishment. Nonetheless, in societies in which corporal punishment is increasingly less prevalent, data show a steady rise in negative attitudes towards physical child discipline (60) and increasing acceptance of legislation against its use (61). As participants in Study 2 indicated, corporal punishment of children is cultural; therefore, attitudes may take longer than the pilot study funding cycle to shift.

The studies presented underscore that attitude measurement is challenging. Attitudes are not always accessible or stable, which affects attitude-behavior consistency (62). In their meta-analysis on attitude-behavior association, Glasman and Albarracín (62) found that easily recalled attitudes that are stable over time best predict behavior, particularly when attitudes are confident. In Study 2, respondents’ attitudes and behaviors remained consistent with predominant cultural views, but with closer inspection, they endorsed conflicting attitudes and beliefs. In this context, it is possible that attitudes towards corporal punishment were not easily recalled (e.g., “*I never really thought about it so much before.”* -Round 2, Cognitive Interview 10). Many caregivers are likely ambivalent about corporal punishment, as evidenced by participants responding “sometimes,” “it depends,” and “maybe” to multiple items. Conflicts in the data may also suggest changing attitudes. A key takeaway from Study 2 was that the cognitive interviewing process seemed to prompt reflection about corporal punishment. Increased thinking about attitudes, and more reporting on attitudes, may increase attitude accessibility (62). A future study may investigate whether administering the ACP with concurrent probing, as was done in Study 2, could serve as an intervention, resulting in an “ACP Heisenberg Principle” or “ACP Mere-Measurement Effect” (63), wherein the act of measurement can lead to positive change.

While attitude measurement is difficult even with valid and reliable questionnaires, these studies identified weaknesses with the ACP itself. First, due to time and resource constraints, cognitive interviewing was not used in the initial development of the ACP. This could have averted issues identified in Study 2, especially around terminology. The original scale did not include several items important to corporal punishment attitudes and behaviors identified in Study 2 (e.g., recency, frequency, and severity of physical punishment; attribution for instances of physical punishment). The SBG research team has since conducted a wider literature review and is pre-testing items informed from Study 2 for inclusion in future iterations of the ACP. A potential issue for scoring is that the ACP queries both attitudes and behaviors, which require separate domain scores that may continue to illustrate conflicting results.

### Measuring for Change

Among ECD researchers, practitioners, and policy makers, there is recognition of the need for data to guide effective implementation and scaling (39). The Measuring for Change movement, with its aspirations for MEL to be dynamic, inclusive, informative, interactive, and people-centered (64), asserts that, rather than focusing on impact alone, ECD programs should use data to generate change, not just measure it.

In aspiring to be “dynamic,” ECD programs should incorporate systems that allow interventionists to adapt to new information in their specific contexts. The SBG team responded to conflicting data (i.e., new information) by conducting a quality assurance study on the ACP, involving participants and other stakeholders in the process (consideration of context). Further efforts to adapt the program based on learning from these studies continues: development and pre-testing of new items for the ACP is underway, and additional measures for corporal punishment behaviors will be considered for future evaluations. Responsive caregiving literature has been reviewed for clarification about safety and connection, and studies of adults raised with and without corporal punishment are underway. SBG will also consider alternative theories of change for the intervention, which achieved the primary outcome of improved neurodevelopment, but not the secondary outcome of behavior and attitude change, at least as measured.

To be “inclusive,” interventions should involve stakeholders in the development and implementation of MEL. More thorough input from Roving Caregivers and other stakeholders with in-depth knowledge about parents’ behavior and the cultural and social relevance of corporal punishment has been sought. Future rounds of questionnaire development, implementation, and evaluation will include this learning.

Lastly, interventions that aspire to be “informative” need data at multiple time points (e.g., development, implementation, and evaluation). A critique of ECD interventions is that data collection traditionally focuses only on child outcomes at intervention’s end (40). SBG’s investigation of corporal punishment attitudes and behaviors is an example of collecting and using data from adults as well as children, and at multiple time points.

### The role of monitoring, evaluation, and learning in the process of scaling

A further consideration regards the use of MEL to inform scaling. The WHO defines scaling as “deliberate efforts to increase the impact of successfully tested pilot, demonstration, or experimental projects to benefit more people and to foster policy and program development on a lasting basis” (65). The current study informed the SBG team’s thoughts on scaling ECD interventions in two key ways.

First, to inform scaling decisions and strategies, data from timely and accurate MEL are needed across different stages of the program. For example, a more detailed assessment of corporal punishment behavior that includes recency, frequency, and severity of punishment may identify smaller changes in behavior than the ACP was able to detect in its original form. Tracking attitudes and behaviors over time can support eventual legislation and normative shifts towards recognition of child rights.

Second, while vertical scaling of ECD interventions is one goal, findings from this case study suggest an alternative scaling construct: one that is chronological and intergenerational. In this case, impact can be achieved as children–raised by adults with knowledge, skills, and self-efficacy to create and sustain safety and social–emotional connections–become parents themselves. Much like the intergenerational nature of corporal punishment behavior, safe environments and strong social–emotional connections could become normative. The question remains whether corporal punishment attitudes and behaviors *will* change alongside improved social–emotional connections over time and in a way that supports lasting change. Demonstrating lasting change provides strong justification for scaling.

Reflecting on measuring for change raised potential approaches to scaling the intervention to other regions. For example, is a focus solely on social–emotional connection sufficient for this postcolonial context, or is more information about the potential harmful effects of physical punishment needed for greater impact? What if attitudes do not change, even if behaviors do? What will we learn by asking about adults’ own experiences of corporal punishment as children? These and other questions illuminate the dynamic, inclusive, and informative potential for measuring attitudes and practices towards corporal punishment, and the potential for a long-term cultural shift in attitudes towards raising children.

The SBG Conscious Discipline intervention is a candidate approach for improving ECD in settings where violence against children is normative. Such violence, including corporal punishment, remains a significant public health problem (66–68). Correlations between physical punishment in childhood and adverse health, behavioral, and neurodevelopmental outcomes across the lifespan (7, 11–13) are a driving force behind efforts to provide alternatives. A valid, sensitive, reliable, and appropriate ACP measure is critical to assess the impact and effectiveness of efforts to shift attitudes and behaviors.

## Data availability statement

The original contributions presented in the study are included in the article/Supplementary material, further inquiries can be directed to the corresponding author.

## Ethics statement

The studies involving human participants were reviewed and approved by St. George’s University Institutional Review Board. Written informed consent to participate in Study 1 was provided by the participants’ legal guardian/next of kin. Study 2 was exempt from IRB review given its focus on measure-testing quality assurance.

## Author contributions

BL is co PI and took the lead in conceptualizing and preparing the manuscript. ET took the lead in conceptualizing and analyzing cognitive interview results. LO conducted interviews and compiled and checked references. RE provided quantitative analyses. TM, LO, LM, RI, and BL conducted interviews and supported analysis. RW is co PI and helped to conceptualize the manuscript. BL, ET, LO, RE, TM, LM, JN, RI, and RW provided critical feedback and assisted with data collection, analysis and writing. All authors contributed to the article and approved the submitted version.

## Funding

The authors declare that this study received funding from the Grand Challenges Canada Saving Brains Programme grant # 0587-03. The funder was not involved in the study design, collection, analysis, interpretation of data, the writing of this article, or the decision to submit it for publication.

## Conflict of interest

The authors declare that the research was conducted in the absence of any commercial or financial relationships that could be construed as a potential conflict of interest.

## Publisher’s note

All claims expressed in this article are solely those of the authors and do not necessarily represent those of their affiliated organizations, or those of the publisher, the editors and the reviewers. Any product that may be evaluated in this article, or claim that may be made by its manufacturer, is not guaranteed or endorsed by the publisher.

## Footnotes

^1^Some literature uses the terms “physical punishment” or “harsh physical punishment” for the legal term “corporal punishment” interchangeably. In this paper we use these terms interchangeably to refer to any form of physical punishment including spanking, pinching, slapping, smacking, whipping, strapping, etc.
