# Supplementary material for: Spare the rod, spoil the child: measurement and learning from an intervention to shift corporal punishment attitudes and behaviors in Grenada, West Indies
Source: Front Public Health. 2023 Aug 29;11:1127687. doi: 10.3389/fpubh.2023.1127687 (PMC10512176; doi:10.3389/fpubh.2023.1127687)
Supplement: Supplementary file 1 [file Image_1.pdf]

Appendix A: Original Attitudes Towards Corporal Punishment Scale

1. What methods do you currently use to get your child to behave?

a. ____

b. ____

c. ____

d. ____

e. ____

2. Which of the listed methods has the most effect on your child's behaviour?

____

3. Have you ever smacked/beaten your child? Yes ☐ No ☐

3a. If *yes*, how recently? Last week ☐ Last month ☐ Last 6 months ☐ Last year ☐

4. Please indicate in which circumstances, if any, you have smacked/beaten your child or children in the past year.

a. ____

b. ____

c. ____

d. ____

5. From this list, which of the following statements comes closest to your personal opinion about smacking/beating your child or children?

a. I think it is always wrong to smack/beat a child, and I won't do it

b. I don't like the idea of smacking/beating a child, but I will do it if nothing else works

c. I'm comfortable with the idea of smacking/beating a child and will do it when I think it's necessary

d. I believe that if you spare the rod, you spoil the child

e. I don’t know

6. Please rate how much you agree or disagree with each of the following statements using the scale provided below.

1 = Strongly agree, 2 = Tend to agree, 3 = Neither agree nor disagree,
4 = Tend to disagree, 5 = Strongly disagree

Item	1	2	3	4	5
6a. Only bad parents smack/beat their children					
6b. Smacking/beating a child is as unacceptable as hitting an adult					
6c. Smacking/beating is a good way of teaching children right from wrong					
6d. The law should allow parents to smack/beat their children					
6e. There is a big difference between smacking/beating a child and physically abusing a child					
6f. There should be a complete ban on parents smacking/beating their children, even as a punishment					
6g. It is sometimes necessary to smack/beat a naughty child					

Please answer the following questions with a “yes” or “no” response.

Item	Yes	No
7. Would you support a law that that made it illegal for parents to use corporal punishment to discipline their children?		
8. Should schools be allowed to use corporal punishment to discipline students?		
9. Is corporal punishment an effective method of disciplining a child?		
10. Does corporal punishment lead to the development of good character?		
11. Does corporal punishment help build respect for authority figures?		
12. Does corporal punishment help children become successful adults?		
13. Does corporal punishment work better than other disciplinary methods that do not involve physical pain?		
14. Do you consider yourself to be a religious person?		

15.) What religion do you follow? ______________________ N/A ☐

Scoring: There is no formal scoring for the ACP as yet.

Groups were compared on individual items using appropriate analysis (e.g., chi square).
