# Supplementary material for: Spare the rod, spoil the child: measurement and learning from an intervention to shift corporal punishment attitudes and behaviors in Grenada, West Indies
Source: Front Public Health. 2023 Aug 29;11:1127687. doi: 10.3389/fpubh.2023.1127687 (PMC10512176; doi:10.3389/fpubh.2023.1127687)
Supplement: Supplementary file 2 [file Image_2.pdf]

Appendix B: HOME-A Acceptance Subscale

II. ACCEPTANCE. This factor covers caregiver acceptance of less than optimal behavior from the child and the avoidance of undue restriction and punishment.

Item	Yes	No
12. No more than one instance of physical punishment during past week.		
13. Caregiver does not shout at child.		
14. Caregiver does not express overt annoyance with or hostility to child.		
15. Caregiver neither slaps nor spanks child during visit.		
16. Caregiver does not scold or criticize child during visit.		
17. Caregiver does not interfere with or restrict child more than three times during visit.		
18. At least ten books are present and visible.		

Scoring: 1 = Yes; 0 = No. Total subscale scoring across all 7 items = 0-7.

Group means were compared using appropriate analysis (t-test).
