# Supplementary material for: Spare the rod, spoil the child: measurement and learning from an intervention to shift corporal punishment attitudes and behaviors in Grenada, West Indies
Source: Front Public Health. 2023 Aug 29;11:1127687. doi: 10.3389/fpubh.2023.1127687 (PMC10512176; doi:10.3389/fpubh.2023.1127687)
Supplement: Supplementary file 3 [file Image_3.pdf]

Appendix C: Revised Attitudes Towards Corporal Punishment Scale

Q0: What's your definition of physical or corporal punishment?

Questions 1-4 are for parents or caregivers only:

Q1: What do you do when your child misbehaves?

____

____

Q2: Which (of those mentioned in Q1) works best?

____

Q2A: Is this the one you usually use? Yes ☐ No ☐
Q2B: If **NO**, which method do you usually use?

____

Q3: [Ask only if not mentioned in Q1]: Have you ever used any form of physical punishment with your child? Yes ☐ No ☐

Q3A: If **YES**: You said in the previous question that you use physical punishment. Please describe (circle one).

With hand ______________________________ (slap, pinch, etc.)

With object _____________________________ (what object(s)?)

Q3B: When is the last time you used physical punishment? (circle one)

Today/this week/past 2 weeks/past month/past 6 months/past year/longer

Q3C: How often do you use physical punishment? (circle one)

More than once a day/daily/more than once a week/once a week/a few times a month/ once a month/ a few times a year/ less than once a year

For the next questions we are going to ask for your opinions about physical punishment; we mean any form of physical punishment, including pinching, pulling, tapping, slapping, spanking, hitting, smacking, giving licks, beating.

Q4: From this list, which of the following statements comes closest to your personal opinion on using physical punishment on your child(ren)? (circle one)

a. I think it is always wrong to use physical punishment on a child.
b. I don't like the idea of using physical punishment, but I will do it if nothing else works.
c. I'm comfortable with the idea of using physical punishment.
d. I believe that if you spare the rod, you spoil the child.
e. Refused, don't know.

The next set of statements asks you to rate how much you agree or disagree with them. Please answer strongly agree/agree/neutral/disagree/strongly disagree.

Q5: Only bad parents use physical punishment on their children. (circle one)

Strongly agree/Agree/Neutral/Disagree/Strongly disagree

Q6: Physical punishment is a good way of teaching children right from wrong.

Strongly agree/Agree/Neutral/Disagree/Strongly disagree

Q7: Using physical force on a child is the same as using physical force on an adult.

Strongly agree/Agree/Neutral/Disagree/Strongly disagree

Q8: There is a big difference between administering physical punishment and physically abusing a child.

Strongly agree/Agree/Neutral/Disagree/Strongly disagree

Q9: There should be a complete ban on parents using physical punishment with their children.

Strongly agree/Agree/Neutral/Disagree/Strongly Disagree

Q10: It is sometimes necessary to use physical punishment with a child.

Strongly agree/Agree/Neutral/Disagree/Strongly disagree

Q11: Schools should be allowed to use physical punishment on children.

Strongly agree/Agree/Neutral/Disagree/Strongly disagree

Q12: Physical punishment is an effective method of disciplining a child.

Strongly agree/Agree/Neutral/Disagree/Strongly disagree

Q13: Physical punishment leads to the development of good character.

Strongly agree/Agree/Neutral/Disagree/Strongly disagree

Q14: Physical punishment helps build respect for authority figures.

Strongly agree/Agree/Neutral/Disagree/Strongly disagree

Q15: Physical punishment helps children become successful adults.

Strongly agree/Agree/Neutral/Disagree/Strongly disagree

Q16: Would you support a law that made it illegal for parents to use physical punishment to discipline their children? Yes/No

Q17: Additional comments (optional)

Scoring: There is no formal scoring for the ACP as yet.

Groups were compared on individual items using appropriate analysis (e.g. chi square).
