# Supplementary material for: Spare the rod, spoil the child: measurement and learning from an intervention to shift corporal punishment attitudes and behaviors in Grenada, West Indies
Source: Front Public Health. 2023 Aug 29;11:1127687. doi: 10.3389/fpubh.2023.1127687 (PMC10512176; doi:10.3389/fpubh.2023.1127687)
Supplement: Supplementary file 4 [file Image_4.pdf]

## Appendix D: Quality Assurance Results for the ACP. Matrix of Original Items, Findings, Modifications, And Item Status.

| Original item (Response option) | Findings from Round 1 | Revised item (Response option) | Findings from Round 2 | Status |
|---|---|---|---|---|
| Definitions and measurement of corporal punishment (behavior) | | | | |
| What methods do you currently use to get your child to behave? (Open-ended response) | - "Currently" is not relevant<br>- Corporal punishment is reactionary, so question should be about what is done when child misbehaves | What do you do when your child misbehaves? (Open-ended response) | - Revision seems clear<br>- There are "degrees" of severity to consider; not all misbehavior is same/warrants the same approach | - How will this item be scored?<br>- Will this be an open-ended or close-ended item? |
| Have you ever smacked/beaten your child?<br>(Yes/No)<br><br>If yes, how recently? (Last week/Last month/Last 6 months/Last year)<br><br>Please indicate in which circumstances, if any, you have smacked/beaten your child(ren) in the past year.<br>(Open-ended) | - "Smack" and "beat" are different<br>- Unclear how to answer if smacked but never beaten<br>- This does not capture other forms of punishment<br>- There is a difference between recency, frequency, and severity of punishment | Have you ever used any form of physical punishment with your child?<br>(Yes/No)<br><br>If yes, please describe. With hand? With object(s)?<br><br>When was the last time you used physical punishment?<br><br>How often do you use physical punishment? | - Revision seems clear, but participants do not always clearly answer each part of the question | - How will this item be scored?<br>- Will this be an open-ended or close-ended item? |

| | | | | |
|---|---|---|---|---|
| There is a big difference between smacking/beating a child and physically abusing a child.<br>(Strongly Disagree/ Disagree/ Neutral/Agree/Strongly Agree) | - "Beating" could be seen as the same as "physical abuse", even if "smacking" is not | There is a big difference between using physical punishment and physically abusing a child.<br>(Strongly Disagree/ Disagree/ Neutral/Agree/Strongly Agree) | - Revision seems clear, though a few participants asked if "big" was necessary ("How to determine what is a big difference?") | - Item should be tested with and without "big" |
| Restrictive question/item or response options | | | | |
| Which of the listed methods (from previous question) has the most effect on your child's behavior?<br>(Open-ended response) | - It depends on the child (behavior, age, etc.) and parent | Which (of those mention in Q1) works best?<br>(Open-ended response)<br><br>Is this the one you usually use?<br>(Yes/No)<br><br>If no, which method do you usually use?<br>(Yes/No) | - Revision seems clear, but participants do not always clearly answer each part of the question | - How will this item be scored?<br>- Will this be an open-ended or close-ended item? |

| | | | | |
|---|---|---|---|---|
| From this list, which of the following statements comes closest to your personal opinion about smacking/beating your child or children?<br><br>(a) I think it is always wrong to smack/beat a child, and I won't do it<br>b) I don't like the idea of smacking/beating a child, but I will do it if nothing else works<br>c) I'm comfortable with the idea of smacking/beating a child and will do it when I think it's necessary<br>d) I believe that if you spare the rod, you spoil the child<br>e) I don't know) | - Options are not mutually exclusive<br>- Multiple statements apply | From this list, which of the following statements comes closest to your personal opinion on using physical punishment on your child(ren)?<br><br>(a) I think it is always wrong to use physical punishment on a child<br>b) I don't like the idea of using physical punishment, but I will do it if nothing else works<br>c) I'm comfortable with the idea of using physical punishment<br>d) I believe that if you spare the rod, you spoil the child<br>e) Refused, don't know) | - Options are still not mutually exclusive (Option B and D and C and D can co-exist)<br>- "Spare the rod, spoil the child" does not necessarily mean severe corporal punishment | - How will this item be scored?<br><br>- How do we resolve this so that the options are mutually exclusive?<br><br>- Do we make each Option its own item? |
| Should schools be allowed to use corporal punishment to discipline children?<br>(Yes/No) | - General sense/purpose of the item seems clear<br>- Participants are not always able to answer "yes" or "no" | Schools should be allowed to use physical punishment to discipline children.<br>(Strongly Disagree/ Disagree/ Neutral/Agree/Strongly Agree) | - Revision seems clear | - Item requires no further revision |

| | | | | |
|---|---|---|---|---|
| Is corporal punishment an effective method of disciplining a child?<br>(Yes/No) | - Participants are not always able to answer "yes" or "no", several answer with "it depends" or "sometimes" | Physical punishment is an effective method of disciplining a child.<br>(Strongly Disagree/ Disagree/ Neutral/Agree/Strongly Agree) | - Revision seems clear, though item may not fully capture situation-dependent use of corporal punishment | - Possible addition of other items may be needed<br>- Possible need to explore alternative response options (never, rarely, half the time, most of the time, always) |
| Does corporal punishment lead to the development of good character?<br>(Yes/No) | - General sense/purpose of the item seems clear<br>- Participants are not always able to answer "yes" or "no" | Physical punishment leads to the development of good character.<br>(Strongly Disagree/ Disagree/ Neutral/Agree/Strongly Agree) | - Revision seems clear | - Item requires no further revision |
| Does corporal punishment help build respect for authority figures?<br>(Yes/No) | - General sense/purpose of the item seems clear<br>- Item considered "too general"<br>- Participants are not always able to answer "yes" or "no" | Physical punishment helps build respect for authority figures.<br>(Strongly Disagree/ Disagree/ Neutral/Agree/Strongly Agree) | - Revision seems clear | - Item requires no further revision |

| | | | | |
|---|---|---|---|---|
| Does corporal punishment help children become successful adults? (Yes/No) | - General sense/purpose of the item seems clear<br>- Definition of “success” may be unclear<br>- Participants are not always able to answer “yes” or “no” | Physical punishment helps children become successful adults (Strongly Disagree/ Disagree/ Neutral/Agree/Strongly Agree) | - Revision seems clear, though definition of “successful adults” may still present an issue | - Item would benefit from additional pre-testing |
| Does corporal punishment work better than other disciplinary measures that do not involve physical pain? (Yes/No) | - General sense/purpose of the item seems clear<br>- Participants are not always able to answer “yes” or “no” | Physical punishment works better than other disciplinary measures that do not involve physical pain (Strongly Disagree/ Disagree/ Neutral/Agree/Strongly Agree) | Item not tested in Round 2? | - Item requires no further revision |
| Theme: Item relationship with construct unclear | | | | |
| Only bad parents smack/beat their children. (Strongly Disagree/ Disagree/ Neutral/Agree/Strongly Agree) | - Most participants disagree with this statement, noting that corporal punishment can be a sign of good parenting, and bad parenting can taken multiple forms<br>- There is some confusion about what is meant by “bad parents” | Only bad parents use physical punishment on their children. (Strongly Disagree/ Disagree/ Neutral/Agree/StronglyStrong Agree) | - Responses are similar to Round 1, and the item seems clear<br>- It is not clear how participants are expected to respond in relation to attitudes towards corporal punishment (i.e., scoring could go either way) | - Item was dropped due to the lack of a clear relationship between the item and the construct |

| Theme: Minor issues with terminology | | | | |
|---|---|---|---|---|
| Smacking/beating a child is as unacceptable as hitting an adult. (Strongly Disagree/ Disagree/ Neutral/Agree/StronglyStrong Agree) | - "Just as bad" used instead of "unacceptable" in practice<br>- Smacking, beating, and hitting all different terms | Using physical force on a child is the same as using physical force on an adult. (Strongly Disagree/ Disagree/ Neutral/Agree/StronglyStrong Agree) | - "Physical force" is too strong a term<br>- General sense/purpose of the item seems clear | - An alternative term for "physical force" needs to be identified and tested |
| The law should allow parents to smack/beat their children. (Strongly Disagree/ Disagree/ Neutral/Agree/StronglyStrong Agree) | - Some confusion about what is meant by "the law"<br>- Some confusion with what this would look like in practice<br>- A few participants selected "neutral" but provided an explanation of disagreement | Item was dropped for Round 2 due to overlap with other items and minor issues identified in Round 1 | N/A | N/A |
| There should be a complete ban on parents smacking/beating their children, even as punishment. (Strongly Disagree/ Disagree/ Neutral/Agree/StronglyStrong Agree) | - Participants did not feel that the qualifier "even as punishment" was necessary | There should be a complete ban on parentings using physical punishment on their children (Strongly Disagree/ Disagree/ Neutral/Agree/StronglyStrong Agree) | - Revision sems clear | - Item requires no further revision |

| | | | | |
|---|---|---|---|---|
| It is sometimes necessary to smack/beat a naughty child.<br>(Strongly Disagree/ Disagree/ Neutral/Agree/StronglyStrong Agree) | - "Naughty" not seen as necessaryremoved | It is sometimes necessary to use physical punishment with a child.<br>(Strongly Disagree/ Disagree/ Neutral/Agree/StronglyStrong Agree) | - Revision seems clear | - Item requires no further revision |
| Would you support a law that made it illegal for parents to use corporal punishment to discipline their children?<br>(Yes/No) | - It is not clear if this item is always understood<br>- Legal/illegal needs to be explained to some participants | Would you support a law that made it illegal for parents to use physical punishment to discipline their children? | - Most participants are able to answer "yes" or/ "no", though a couple responded "maybe" or did not provide an answer<br>- There were some qualifying statements to explain responses that might be lost with a single close-ended question | - Under-standing of legal/illegal as part of this item will need to be tested in a population similar to the target population |
